# Valproate-Induced Bicytopenia: A Case Study

**DOI:** 10.7759/cureus.22327

**Published:** 2022-02-17

**Authors:** Varun Jaitpal, Sushil Gawande

**Affiliations:** 1 Psychiatry, NKP (Narendra Kumar Prasadrao) Salve Institute of Medical Sciences and Lata Mangeshkar Hospital, Nagpur, IND

**Keywords:** clinical case report, thrombycytopenia, leukopenia, bicytopenia, valproate

## Abstract

This case report describes a 22-year-old male patient diagnosed with schizoaffective disorder. A multidrug regimen including olanzapine and lorazepam was initiated for the patient. Sodium valproate was added to the regimen in due course and it was associated with bicytopenia in the form of thrombocytopenia and leukopenia. Valproate was identified as the offending drug and further doses were withheld. Lithium was used as a substitute to valproate and the patient spontaneously recovered without any further complications. This case report highlights the necessity of periodic investigations and frequent logging of blood indices to counter the threat of fatal adverse drug reactions.

## Introduction

Recent advances in medical science and technology have ensured the availability of a multitude of treatment regimens to counter the burden of schizophrenia spectrum disorders [1]. Even though antipsychotic medications are the preferred line of management, mood stabilizers like valproate as well as lithium are commonly used in addition to second-generation antipsychotics for treating the mood symptoms that often accompany the negative and positive symptoms of schizophrenia and schizoaffective disorders [2,3]. The utility of valproate in treating mood symptoms and disorders was explained by Lambert et al. in 1966 and has been used in the acute and long-term management of these disorders ever since [4].

Extracted from the plant of *Valeriana officinalis*, valproic acid (also known as valproate) is a derivative of valeric acid [5]. Available as sodium valproate, this drug has multiple mechanisms of action. Increasing the concentration of gamma-aminobutyric acid (GABA) by preventing its disintegration along with blocking the voltage-gated calcium, potassium, and sodium channels is of prime importance in its use as a potent drug. Valproate also inhibits histone deacetylase (HDAC1 in particular) leading to up-regulation of genes regulating apoptosis and antitumor action. The expression of various genes regulating survival of the cell, transcription, transduction of signals, ion balance, etc. is also modified by valproate [6].

Side-effects commonly associated with valproate are tremors, fatigue, disturbances of the gastrointestinal system, weight gain, etc. Adverse reactions which could prove to be life-threatening include disturbances in the endocrine, neurological (excessive sedation), metabolic and hematological (thrombocytopenia) systems [4]. Valproate-induced leucopenia, particularly neutropenia, has also been reported in the past [7].

Extensive research has been done in the field of pancytopenia while the territory of bicytopenia remains relatively uncharted [8]. A diagnosis of bicytopenia can be made when there is a reduction in two out of three cell lineages namely: red blood cells (RBCs) or erythrocytes, white blood cells (WBCs), or leucocytes, and platelets or thrombocytes [9]. The etiology for bicytopenia/pancytopenia ranges from marrow suppression to malignant infiltration resulting in an increase in the destruction or decreased production of cells [9,10]. An increase in the pooling of these cells in the organs such as the spleen and peripheral destruction of cells by toxins also form an important etiology for bicytopenia/pancytopenia [9].

Through this case report, we aim to demonstrate the occurrence of valproate-induced bicytopenia. Principally, it consolidates the evidence for frequent investigations, examination, and monitoring of blood indices to counter the threat of potentially fatal drug reactions.

## Case presentation

In November of 2021, a 22-year-old male was brought to the emergency department of our hospital by his parents for recent bizarre behavior for the past few weeks in the form of singing and dancing without provocation and poor self-care alongside talking big and sleep disturbances. On eliciting a history, it was found that the total duration of illness was three years with a history of behavioral disturbances in the form of suspiciousness, hearing of voices inaudible to others, wandering away episodes, and multiple deliberate self-harm attempts. In the past, the patient had received antipsychotics with improvement. However, poor compliance with pharmacotherapy was noted. The patient had also received electroconvulsive therapy (ECTs) one month ago with minimal improvement. At the time of presentation, the patient was not taking any psychotropic medication. His initial psychiatric evaluation revealed a disheveled appearance, grandeur, self-reported decreased need for sleep, and impaired judgment. Total denial of the illness indicated poor insight. Based on the history and presenting symptoms, a diagnosis of schizoaffective disorder, bipolar type, multiple episodes, currently in acute episode was made as per the* Diagnostic and Statistical Manual of Mental Disorders, Fifth Edition (DSM-5)* criterion [11]. As the patient was unmanageable at home, he was admitted to the inpatient facility of the Department of Psychiatry for further management and evaluation.

On admission, a complete blood profile of the patient was done and after the tests returned within normal limits for hematological, hepatic, and renal functions, a second-generation antipsychotic, olanzapine, was chosen as the primary antipsychotic. Olanzapine was administered orally and titrated up to 7.5 mg tablets twice a day (total 15 mg/day). A benzodiazepine in the form of lorazepam (2 mg in the night) was started to regulate sleep. Olanzapine was gradually titrated to a combined dose of 20 mg/ day over the next week. As the patient did not show adequate improvement on these doses, another antipsychotic was added to the regimen on day 10. Trifluoperazine (a first-generation antipsychotic) was added at 10 mg/day (two equally divided doses). This was titrated to 20 mg/day by day 16. Lorazepam was tapered to 1 mg/day by day 16 and the three-drug regimen was continued for seven days. Regular blood counts were done and no abnormality was noted until day 23 of admission. Inadequate response to this regimen and persistence of affective features prompted the introduction of sodium valproate as an adjunct in two equally divided doses of 300mg (total 600 mg/day) on day 24 which was titrated up to 800 mg/day (two equally divided doses of 400 mg) and then to 1000 mg/day (two equally divided doses of 500mg/day) by day 30. Lorazepam was omitted by day 30. Trifluoperazine was also tapered and omitted by day 30. Since the patient started showing signs of improvement of affective features with the introduction of sodium valproate, it was further increased to 1200 mg/day (two doses of 600 mg each) on day 40. As per the regular monitoring schedule, a routine complete blood count (CBC) investigation was done on day 42 and this report indicated, leucopenia (total leucocyte count: 3200 cells per microliter) and thrombocytopenia (platelet count: 73,000 cells per microliter). A repeat sample was sent to rule out any errors and yet the results obtained were consistent with the initial finding. A physician's opinion was taken and a probable diagnosis of drug-induced bicytopenia was made after consultation. Sodium valproate was reduced to 1000 mg/day as it was well tolerated earlier. However, the bicytopenia further worsened on day 44 with the leucocyte counts dropping to 3000 cells per microliter and the platelet count dropping to 27,000 cells per microliter. Physical examination of the patient did not reveal any bruises, petechiae, or lymphadenopathy. Valproate was completely withdrawn herewith and was substituted with lithium 900mg/day (three equally divided doses). Regular CBC reports post-withdrawal of valproate showed a gradual increase in the blood counts. The leucopenia was resolved by day three of withdrawing valproate while thrombocytopenia was resolved seven days post stopping the drug. Hydration of the patient was maintained throughout.

Due to a poor response of psychotic symptoms, even to a combination drug therapy given for a sustained duration of time, clozapine was chosen for further management. Clozapine 25mg/ day was started on day 55 of admission and was titrated to 300 mg/day by day 69 of admission. The patient was finally discharged on day 75 on clozapine, lithium, and olanzapine, with a plan to omit olanzapine on further follow-ups.

## Discussion

Valproate as a drug requires prudent monitoring including liver function and complete blood count tests at baseline followed by frequent monitoring especially during the first six months of therapy. Serum drug levels should be monitored (therapeutic range: 50 to 125 mcg/ml). Contraindications for the use of valproate include hepatic impairment, hypersensitivity, pregnancy, disorders of the mitochondria and urea cycle, renal impairment, myelosuppression, etc. [6]. 

Thrombocytopenia, statistically, is diagnosed as a platelet count of <150x109/L and becomes critical when the count becomes <50000 per microliter [4,12]. Several etiologies ranging from hemodilution, bleeding causing consumption, and exposure to drugs such as valproate and heparin have been listed for thrombocytopenia [12]. Drug-induced thrombocytopenia (DITP) may be due to the direct toxic effect of the molecule on platelets leading to dysfunctional production within the marrow or increased peripheral destruction of megakaryocytes in circulation increasing the threat of fatal hemorrhages [13]. Management of DITP principally includes the withdrawal of the offending drug followed by replacement if required. Platelet transfusion proves to be futile in these cases as the drug molecules or metabolites present in the plasma may cause platelet destruction [13].

Leukopenia can be defined as an absolute decrease in the number of circulating WBCs below the normal levels [14]. Statistically, a total WBC count <4300 per microliter is diagnosed as leukopenia [15]. The mechanism of drug-induced leukopenia is thought to be suppression of the bone marrow and direct inhibition of the marrow progenitor cells. A quantitative or qualitative defect in WBCs predisposes the patient to infections, which can prove to be fatal [14].

As mentioned earlier, bicytopenia is the reduction in the cells of two cell lineages and can therefore be of three types. In a study conducted by Singh et al., anemia with thrombocytopenia was established as the commonest type seen in 61% of patients. On the other hand, leukopenia accompanied with thrombocytopenia was deemed to be the rarest type and was seen in only 13% of participants [8,9]. 

As it is evident from our case report, our patient fell into the rare category of a combination of leukopenia and thrombocytopenia following the initiation of valproate therapy. As per the Naranjo adverse drug reaction probability scale, it is probable that valproate was the cause of our patients’ bicytopenia [16]. Literature has earlier implicated olanzapine in the case of neutropenia [17], but the temporal relationship between starting of valproate therapy and the onset of bicytopenia within weeks of initiation alongside the spontaneous resolution of bicytopenia following the withdrawal of the drug despite the continuation of olanzapine further consolidates the theory of valproate-induced bicytopenia. Only a handful of articles highlighting the aforementioned association has been put up to date. This case report magnifies the importance of frequent investigations and monitoring of the drug dose and effect on blood indices to prevent fatal adverse effects. 

## Conclusions

Drug-induced bicytopenia is a rare but potentially fatal event and can be diagnosed with the help of a temporal relationship of decline in blood indices following exposure to the offending drug after the exclusion of other possible etiologies. Pinpointing this etiology and prompt withdrawal of the offending drug is of utmost importance to prevent fatal outcomes. Frequent investigations and periodic charting of blood counts play a critical role in monitoring the effects of the drugs. Prevention or prompt treatment of such adverse reactions form a vital cog in ensuring the patient wellbeing and ensure good compliance contributing towards a good prognosis. This case study also stresses the need for more detailed studies directed towards the etiology and management of bicytopenia.
